# Security Techniques for Prevention of Rank Manipulation in Social Tagging Services including Robotic Domains

**DOI:** 10.1155/2014/832638

**Published:** 2014-07-09

**Authors:** Okkyung Choi, Hanyoung Jung, Seungbin Moon

**Affiliations:** ^1^Department of Computer Engineering, Sejong University, Seoul 143-747, Republic of Korea; ^2^Department of Knowledge Information Engineering, Graduate School of Ajou University, Suwon 443-749, Republic of Korea

## Abstract

With smartphone distribution becoming common and robotic applications on the rise, social tagging services for various applications including robotic domains have advanced significantly. Though social tagging plays an important role when users are finding the exact information through web search, reliability and semantic relation between web contents and tags are not considered. Spams are making ill use of this aspect and put irrelevant tags deliberately on contents and induce users to advertise contents when they click items of search results. Therefore, this study proposes a detection method for tag-ranking manipulation to solve the problem of the existing methods which cannot guarantee the reliability of tagging. Similarity is measured for ranking the grade of registered tag on the contents, and weighted values of each tag are measured by means of synonym relevance, frequency, and semantic distances between tags. Lastly, experimental evaluation results are provided and its efficiency and accuracy are verified through them.

## 1. Introduction

As social networking services are becoming common and robotic technology has advanced, the number of social tagging services for various applications including robotic domainsis also rising. A tagging service allows a user to enter a description of the contents of a resource using a simple term or an annotation. Social tagging, also referred to as collaborative tagging, has a flat structure unlike the taxonomy which has a systematic structure by specialists in a specific domain and can be also called a collection of tagging data formed in an open environment.

The term tagging refers to the action of entering a keyword or a tag, namely, a search label that can represent contents. It contains a keyword so that a site administrator can easily classify the contents into categories by subject. Tagging can also be used for a link to blogs or webpages. As the tags are classified and arranged based on subjects or categories, general users can use the tags to gain access to corresponding content [1]. Though tagging plays an important role when users are finding the exact information through web search, reliability of semantic relation between web contents and tags is not considered. Spams are making ill use of this aspect and put irrelevant tags deliberately on contents such as photos, videos, and blogs and induce users to advertise contents when users click items of search results. As the number of users utilizing social media services is increasing, many researches have proposed the methods focusing on social recommendation services with collaborative tagging [2–8]. In common collaborative tagging systems such as https://Delicious.com, http://Twitter.com, http://Facebook.com, and https://www.Flickr.com, users freely assign keywords or tags to annotate the contents, such as movies and pictures for sharing purposes. But, traditional collaborative tagging systems do not consider the semantics of tags [2], so ambiguous tags can be used for representing contents. To solve the problem of lack of relationship between tags, Kim et al. [2] proposed a semantic collaborative filtering method for enhancing the quality of recommendations and Kleinberg [3] addressed the central issue of the distillation of broad search topics through the discovery of “authoritative” information sources on the topic and developed HITS (hypertext induced topics search) algorithm. The HITS algorithm made use of authorities and hubs to serve the efficient and accurate ranking [3]. Wang et al. [9] introduced a framework for the personalization of social media systems and Hotho et al. [8] presented FolkRank, which is the leading one among social tagging service algorithms to solve the problem of the existing method of PageRank. As stated so far, user-entered tagging information can be utilized to draw out more accurate search results, because it is capable of depicting and summarizing the webpage contents in more detailed and precise manners than extracted keywords.

Though some social tagging services were proposed in past researches, problematic points can be summarized as follows. First, traditional collaborative tagging systems allow anyone to freely post information. Therefore, they can lead to unnecessary and unwanted tags that are completely irrelevant to the contents of a webpage. Second, a growing number of malicious websites take advantage of such loopholes and post tags that do not match up with the contents of a webpage in order to increase search engine ranking. Third, the graph-based previous work does not take into consideration the synonym relevance, frequency, and semantic distances between tags.

Thus, the purpose of this study is to enable effective ranking services by analyzing the algorithms from previous studies and by developing a new method for blocking out unsuitable tagging methods to prevent the use of personalized social ranking services as for rank manipulation. To address the discussed issues, ranking measurement algorithm is proposed to rank the grade of registered tag on the contents, and weighted values of each tag are measured by means of synonym relevance,, frequency, and semantic distances between tags. In addition, it is designed along with step-by-step designing method for efficient accurate search and composition and its efficiency and accuracy are verified by comparison with the existing systems.

The rest of this paper is organized as follows. Several related works are compared in the next section. In Section 3, the design of the suggested method is described, along with the principles of its modules and advanced algorithm. In Section 4, we describe experimental results to verify efficiency and validity of our research. Conclusions are provided in Section 5.

## 2. Related Work

### 2.1. Social Tagging Systems

Social tagging is the practice of allowing any user to freely annotate the content of a webpage with arbitrary keywords [2, 3]. Social media sites with social tagging have become tremendously popular in recent years. Social tagging changes people's life patterns and also gives a rich distribution of information. Therefore, recommender systems that are based on social tagging have become an active and growing topic of studies. These studies can be divided into three areas: tag suggestions, social searches, and social recommendations [2].

Kim et al. [2] proposed a semantic collaborative filtering method for enhancing the quality of recommendations that are derived from user-generated tags. In addition, they explore several advantages of semantic tagging as a means for avoiding ambiguity, synonymy, and semantic interoperability, which are notable challenges in information filtering. The proposed approach uses social tagging to find semantically similar users and subsequently discovers semantically relevant items for each user.

In collaborative tagging systems such as https://Delicious.com and https://www.Flickr.com, users assign keywords or tags to their uploaded resources such as bookmarks and pictures for sharing purposes. The collection of resources and tags generated by a user is called a personomy and the collection of all personomies constitutes a folksonomy. The most significant purpose of a folksonomy is to help users to find useful resources or experts on specific topics in an efficient way [4].

Wang et al. [9] introduced a framework for the personalization of social media systems. Their study consists of three tasks that would benefit from personalization: collaborative tagging, collaborative browsing, and collaborative searching. They propose a ranking model for each task that integrates the individual user's tagging history in the recommendation of tags and content in order to align its suggestions with the individual user's preferences. They use two real datasets to demonstrate all three tasks. The personalized ranking should take into account both the user's own preferences and the opinions of others.

### 2.2. Graph-Based Ranking Algorithm

For the web, where documents are linked to each other with hyperlinks, methods such as PageRank and HITS have been developed for calculating a criticality score by analyzing the link structure between the documents using large-size web graphs. According to Google's PageRank method that was developed in 1998, the more a webpage is referred to by other webpages, the higher the criticality score is [4]. Existing search engines determine searching ranks based on keywords, but Google gives searching ranks based on the number of pages that are referred for each webpage. It has the advantage of bringing more accurate search results and a higher quantity of search results as compared to other search engines. Therefore, it is the most used search engine in schools and research centers, because its searching rank decision method differentiates it from other search engines. However, it has some disadvantages in that it sometimes returns broken links or information that has nothing to do with the user's intentions. Furthermore, Google's PageRank method can result in a “Google Bomb.” The composite score that determines an overall rank in Google is calculated by summing the PageRank score, which gives higher scores to webpages that have more linkage, and the content score, which gives higher scores based on the criticality of the text. However, this method has a weakness. The criticality of a webpage can be purposely elevated by a malicious program, such as Link Farms, that uses spam pages in order to raise the searching rank of a specific page.

Kleinberg [3] addressed the central issue of the distillation of broad search topics through the discovery of “authoritative” information sources on these topics. They proposed and tested an algorithmic formulation of the notion of authority that is based on the relationship between a set of relevant authoritative pages and the set of “hub pages” that join them together in the link structure. Their formulation is associated with the eigenvectors of certain matrices associated with the link graph. These associations in turn motivate additional heuristics for link-based analysis.

In Noll and Meinel's study [11], social annotation via the so-called collaborative tagging is the process by which many users add metadata to shared content in the form of unstructured keywords. In their paper, they analyzed large sets of real-world data in order to explore and study social annotation and tagging with regard to their usefulness for web document classification. They were interested in finding out which kinds of documents are annotated more by end users than others, how users tend to annotate these documents, and, in particular, how these user-generated folksonomies are compared with the top-down taxonomies that are maintained by classification experts for the same sets of documents. They described what could be deduced from the results for further research and development in the areas of document classification and information retrieval.

FolkRank, which was presented by Hotho et al. [8], is the leading ranking algorithm among social tagging service algorithms. This ranking algorithm, in general, conducts a structural analysis of the graph (network) link/connection. PageRank basically measures the importance of a webpage using a probability-based method that is based on an analysis of the connection of hyperlinks [4, 7, 8]. FolkRank also added additional functionality to the existing PageRank method by means of a ranking algorithm that analyzes the relation between links to a folksonomy in a graph-based approach but failed to present ranking results in a more detailed and accurate manner by analyzing the mere relationship between users, tags, and resources. Another disadvantage is that users may intentionally write tags and expose advertisements for wrongful purposes. So, it is necessary to precisely extract a keyword representing the actual document.

## 3. Proposed Method

Collaborative tagging systems help voluntary users allocate tags freely to a great number of resources that are available on websites. Folksonomies are sets of resources that are collected and tagged by different users and classified in a bottom-up manner. A folksonomy is comprised of the users, resources, tags, and tag allocation relationships between them [4]. The HITS algorithm made use of authorities and hubs to give ranking scores where authority scores increase as they are linked more by the webpages of major websites, with herb scores higher when linked more by the webpages of other major websites [5]. The Google PageRank algorithm and Kleinberg's HITS algorithm adopt a method of determining the ranking based on the number of pages referred for each webpage and the number of hyperlinks. This method is not suitable for resource-based social network services such as Facebook or Twitter.

Li et al. [6] gave a comparative analysis of the tags created by users on a webpage that was bookmarked on “delicious” and the keywords that were extracted automatically from the contents of identical webpage. Their analysis showed that user-generated tags are sufficient for describing the contents of webpages. In addition, they conducted an experimental analysis that concluded that tags provide a description and summary of the contents that are based on a human perspective, rather than only a set of extracted keywords [6, 7]. But, the problem is that a lot of noisy data are also introduced as the tags and they are freely entered by users without any restrictions.

This paper, therefore, aims to propose research methods and solutions for the issues indicated above. First, an analysis will be conducted on the types of ranking manipulation in social tagging services. This will be followed by suggestions for improved methods for detecting ranking manipulation that are suitable for the present environment for social tagging services. Second, an improved ranking algorithm will be proposed to provide more accurate and reliable information. Features of a ranking method will be investigated through the analysis of the existing algorithm for social tagging services.

### 3.1. Proposed Method

The most important reason to use tags is their inherent simplicity. By inputting a few words, users can assemble a large collection of tags in a minimal amount of time. Social tagging is also flexible in a way that is not limited by situation or purpose. However, there are some spammers who utilize tagging services for antisocial behavior. Since spammers can influence tagging systems and post spam using scripts, they attack again when their spam is eliminated. Even when spam protection techniques are used, spammers find another way to attack. Therefore, it is difficult to prevent spam completely.

Smith [10] suggested several methods for fighting spammers. The first one is to prevent automatic tagging by checking whether the tagging is being executed by a human or a bot. CAPCHA (Completely Automated Public Turing Test for Telling Computer and Humans Apart) is an automatic tagging protection technique that can protect tagging from simple spam texts. The second one is to differentiate user rights so that systems can block the access of a user who enters spam tags. This method cannot prevent spam tagging completely, but it can minimize its impact. The third one is a method for deleting or putting a special mark on a relevant tag when another user reports that the relevant tag is inappropriate.

**Figure 1:**
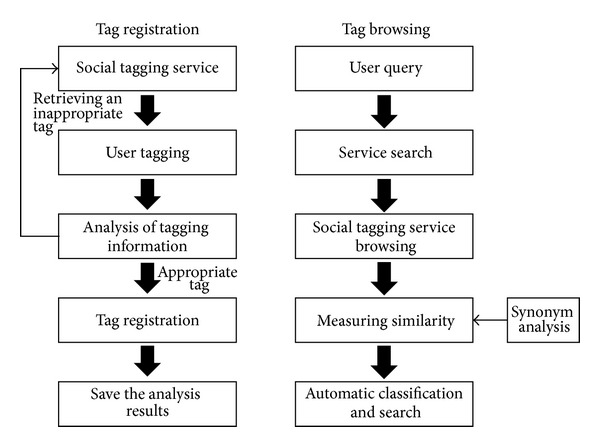
Overall proposed method.

The methods include an automatic tagging protection function so that some inappropriate tagging can be blocked, but there is a problem. Synonyms or similar words cannot be recognized. Since they do not include any analysis on similar tags or relations between tags, there is an advantage that tags even substantially significant can be excluded. For example, as for a specific resource, “folksonomy” is recognized as a correct tagging but “collaborative tagging” is recognized as a wrong one. Such an error is caused by the absence of analysis on tags for synonyms or similar words. Therefore, this study analyzes synonyms or similar-word tags in order to enable more reliable and efficient social tagging service registration and browsing.  Figure 1 is overall proposed method. The left side of Figure 1 shows the process of registering tags using social tagging service and the right side of Figure 1 shows the process of searching the reliable and exact social tagging service when a user queries a tag.

**Figure 2:**
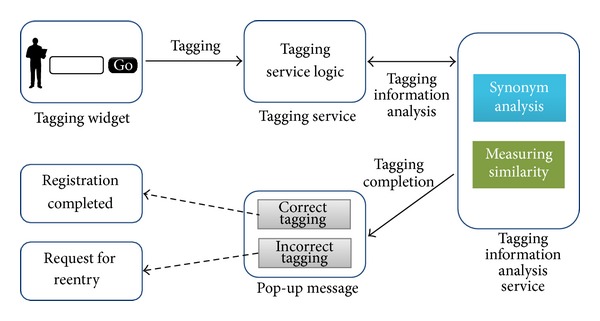
Process of tag registration.

More detailed process is shown in Figure 2.

Figure 2 shows the tag registration flow and its phased process is as follows.

1. The user inputs a tag.
2. The input tag goes through the tagging service phase and then it continues to the synonym analysis and similarity measuring process using the tagging information analysis service.
3. The tagged information is judged whether it was a correct or a wrong tag through tagging information analysis service and returns the result.
4. If it is a correct tag, it is registered in the social tagging service with a message that says [Registration completed], but if not, it requests the user to retry.

The previous works are about finding the social tagging services which only have the tags that are simply registered, but this study is different given the following points. First, if a user makes a query in order to find a social tagging service, it analyzes the words from top to bottom in a web page and extracts the words similar to the tag that the user entered. Lastly, based on established Ontology, the extracted words and user's queries are analyzed in order to compare the relevance and similarity between them. It then measures the level of similarity. As a result, they are automatically classified according to the level of similarity and then return the results which have the highest similarity to the user. Therefore, search results based on automatic classification can be more reliable and accurate.

### 3.2. Advanced Semantic Tagging Algorithm

The key to advanced semantic tagging algorithm lies in how precisely the quality of resources and an individual's interest are reflected in the result. The weighting method that is proposed in this study is based on users, resources, tags, and relations, where different return values are given for the same content if users have different interests.

Equation (1) represents an advanced semantic tagging algorithm for automatically classifying and authorizing the ranking of social network documents using semantic metadata. This algorithm consists of the cosine similarity of the existing vector model and the reflected value proportional to the added weight (*k*_*j*_) proposed in Definition 1 [12–14]. Consider

(1) $$\begin{array}{c} \text{sim}\left(d_{j}q\right)=k_{j}\times \frac{{\vec{d}}_{j}\cdot \vec{q}}{\left|{\vec{d}}_{j}\right|\times \left|\vec{q}\right|}. \end{array}$$

This unit describes the reflected value proportional to the added-weight for application to the ranking measurement algorithm. The reflected value of weight (*k*_*j*_) for the automatic classification and ranking is as follows. Definition 1 is the reflected value of weight (*k*_*j*_) based on *R*_*j*_, a variable measuring the synonym relation between each tag (*j*) of social tagging information; *D*_*j*_, a variable measuring the connection relation between each tag; *U*_*j*_, a variable measuring the connection relation between users and tags; and *Re*_*j*_, a variable reflecting the importance of resources.

**Definition 1 .**

Consider

(2) kj=RjDj×Uj×Rej,

where *R*_*j*_ is a variable measuring the synonym relation between each tag, *D*_*j*_ is a variable measuring the connection relation between each tag, *U*_*j*_ is a variable measuring the connection relation between users and tags, and *Re*_*j*_ is a variable reflecting the importance of resources.

Definition 2 is a* semantic relevance value *(*R*_*j*_) based on *f*_*ij*_, a variable measuring the number of tags (*j*) generated in content (*i*) and *S*_*r*_, a variable measuring the similarity (synonym relation) between each tag.

**Definition 2 .**

Consider

(3) $$\begin{array}{c} R_{j}=\frac{f_{ij}}{T_{j}}\times S_{j}, \end{array}$$

where *f*_*ij*_ is a variable measuring the frequency of tags (*j*) registered in contents (*i*), *T*_*j*_ is a variable measuring the total number of tags (*j*), and *S*_*j*_ is a variable measuring the similarity (synonym relation) between each tag. Consider

(4) $$\begin{array}{c} S_{j}=\frac{1}{\sum_{i=1}^{n}\left(S_{r}\right)},\;\left(0<\left(S_{r}\right)\leq 1\right), \end{array}$$

where *f*_*ij*_ is a variable measuring the frequency of tags (*j*) registered in contents (*i*) and *T*_*j*_ is a variable measuring the total number of tags (*j*). If the tag “amazon” was registered 7 times in contents (*i*), similarity “0.7” is measured for variable *S*_*j*_; if the tag “tablet” is registered twice in contents (*i*), similarity “0.2” is measured for variable *S*_*j*_. If the frequency of the tag is “0”, then the value “0.001” is measured for variable *S*_*j*_; if the frequency of the tag is over “10”, then the value “1.0” is measured for variable *S*_*j*_.

Definition 3 is the relation between each tag; that is, a variable *D*_*j*_ defines the proximity depending on a distance. A variable (*D*_*j*_) measuring the relation between each tag determines the weight with the use of the proximity (*H*_*p*_) between each of the parallel nodes and the proximity (*V*_*p*_) between each of the vertical nodes in the structures of each content.

**Definition 3 .**

Consider

(5) $$\begin{array}{c} D_{j}=\overset{n}{\underset{i=1}{\sum }}\left(H_{p}\times V_{p}\right), \end{array}$$

where *H*_*p*_ is the horizontal proximity between each tag and *V*_*p*_ is the vertical proximity between each tag.

The semantic distance variable *D*_*j*_ uses the proximity between each horizontal node (*H*_*p*_) and the proximity between each vertical node (*V*_*p*_) of each of the contents' structures.

### 3.3. System Architecture

**Figure 3:**
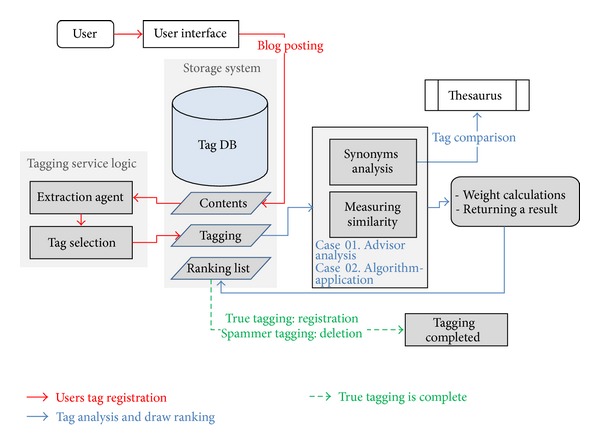
System architecture.

Figure 3 is the system architecture of the suggested algorithm. The evaluation was conducted in a passive and automatic manner, analyzed by an administrator, and applied to the system using the algorithm.

When a user posts information on the bulletin board, it is saved in the “Contents” table of the storage system. The extraction agent retrieves the tags from the “Contents” table and shows the tags to the user. When a user clicks the Save button after selecting tags, the user's tagging registration is completed and the tags are stored in the “Tagging” table. A thesaurus classification method is used to compare the synonym relevance of each registered tag and to obtain a weighted value for each tag by measuring the similarity between the contents and the tags. A weighted value for each tag is stored in the “Ranking List” table in the Tag DB. The weighted values of the stored tags are used to classify normal tags and spam tags. The spam tags are deleted based on the results from the “Ranking List.”

### 3.4. System Flowchart

**Figure 4:**
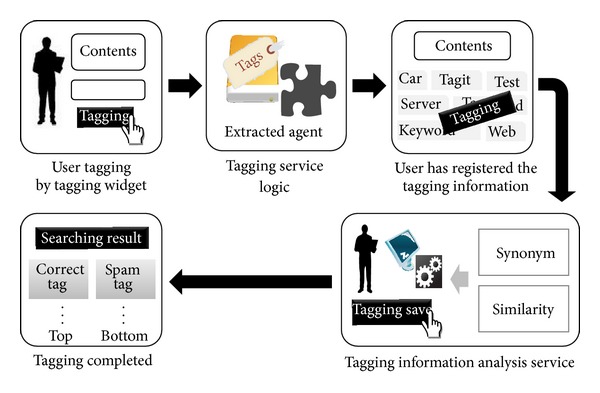
System flowchart.

Figure 4 is a flowchart of the suggested method. It is a detection process based on a user's tag registration and suggestion of contents.

The phased process is as follows.

1. The user posts information on the bulletin board.
2. In the logic phase of the tagging service, the program selects the tags that are automatically extracted from the existing contents of the webpage.
3. The user can select tags from the tag lists that have been extracted automatically or the user can register a tag by typing it in.
4. Once the new tag has been entered, the tag goes through the tagging service phase. This phase includes synonym analysis and the similarity measurement process using the tagging information analysis service.
5. After the application of the algorithm using synonym analysis and the similarity measurement process, valid and invalid tags can be distinguished from one another.
6. If the new tag is a valid tag, the tag is registered in the social tagging service and the following message is displayed: [Registration Completed]. However, if the new tag is invalid, the program asks the user to attempt the registration process again.

## 4. System Implementation

### 4.1. Implementation Environment

The program was developed using HTML, PHP5, Javascript, and Ajax based on the CentOS operating system and the Apache Web Server. The database uses MySQL. The implementation environment of server is as shown in Table 1.

### 4.2. Implementation Results

#### 4.2.1. User Screen

**Figure 5:**
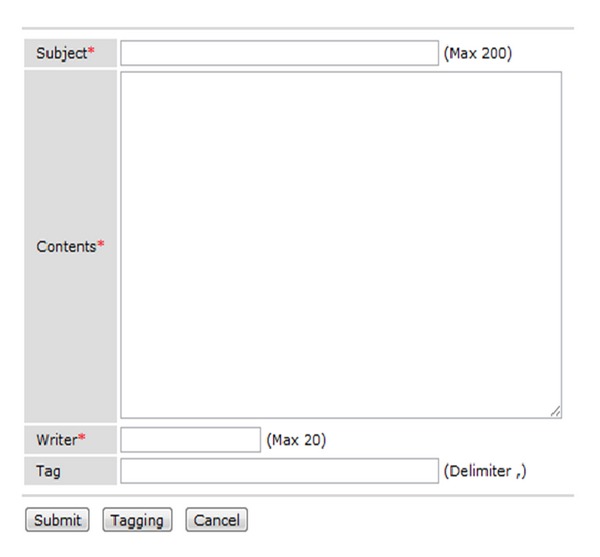
User input screen.

**Figure 6:**
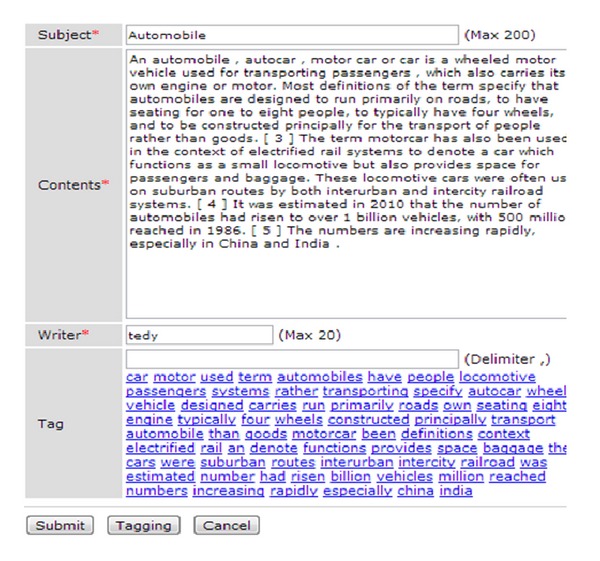
User input screen (tags that are extracted automatically are shown in this screen).

Figure 5 is a user input screen for entering written words in a message board and typing in tags. As shown in Figure 5, after entering information, the user clicks the [Tagging] button and extraction tags related to the topic are displayed as shown in Figure 6. When the user clicks the tag related to his/her topic, it is registered. As for common message boards, when the user clicks the “Submit” button, the information in the written message is transmitted to the server and saved in the MySQL database.

Figure 6 shows the automatically extracted tags that can be used as tags when the user clicks the “Tagging” button. The user can choose a tag that is related to the existing subject and contents of the webpage. Tags can also be entered manually by the user.

**Figure 7:**
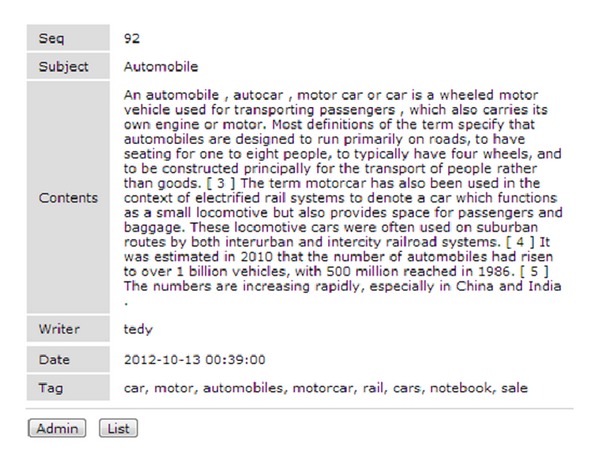
User input screen (tags are completed in the bulletin board).

When the user clicks the “Submit” button, the user's tags are saved in the database and they can be displayed in the Bulletin list as shown in Figure 7.

**Figure 8:**
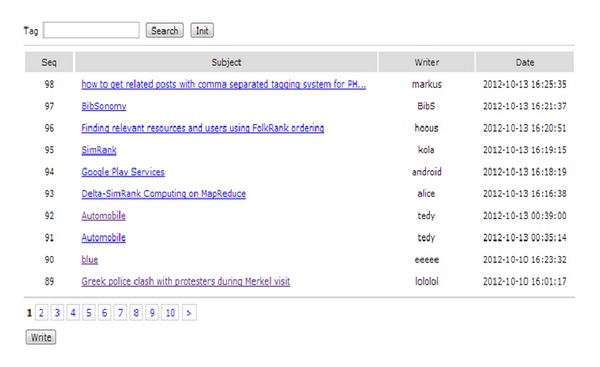
Bulletin list written by a user.

Figure 8 shows a bulletin list that was created by a user. The user's postings are saved on the website. The user can see the content by just clicking on the title of a list. If the user wants to search based on a specific keyword, the meaning of a search keyword that is used within the domain can be defined accurately by an ontology server. Additionally, based on the constructed ontology, similar words are searched for and interpreted [15]. In this way, the user can see the exact results that he/she is searching for.

#### 4.2.2. Administrator Screen

**Figure 9:**
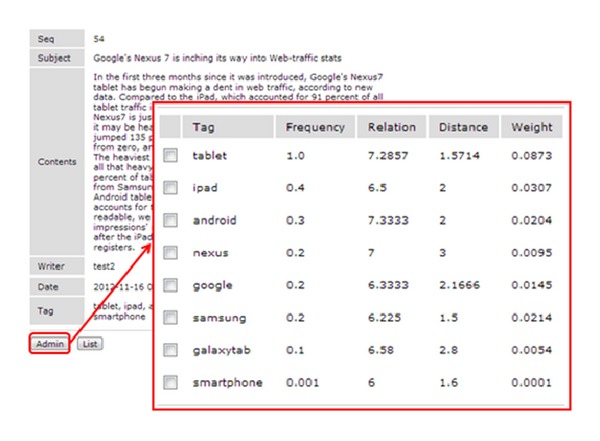
Administrator screen.

The administrator can identify the weighted value of the tags that were written by the user. The administrator can delete a tag by making judgments regarding valid tagging and spammer tagging based on the result of the weighted value shown in Figure 9.

## 5. Performance Evaluation

In this study, an experiment was conducted for four weeks after credible statistics were secured from preoperations. It was conducted with 18 graduate students who had been attending the University for several weeks and who had enough knowledge about how to use the system.

For the analysis of the performance of the proposed system, real data was taken and used for comparison. To evaluate the degree of similarity between the tag and the measuring methods of synonym analysis, this study used actual dataset contents that were suggested beforehand in the blog. This study was executed using Android-based platforms such as Nexus S and Galaxy S3. The evaluation test was conducted in two ways to prevent the experiment from ranking in manipulation on social tagging. One test method was to measure the simple frequency of blog contents and registered tags and the other was to measure tag similarity and perform synonym analysis. Through these methods, judgments were made about whether a tag was valid or invalid. Tagging refers to methods for assigning one or several tags to one piece of content.

### 5.1. Comparison with Existing Studies and Suggested Method

**Figure 10:**
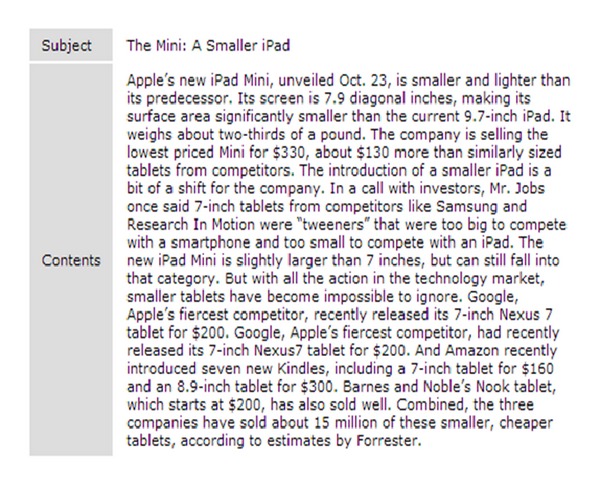
Resource of a test object.

For this experiment, we posted the contents related to title “The Mini: A Smaller iPad” on the bulletin board. Tags were registered by using the automatic tag extraction system in the blog site.  Figure 10 shows what was written about the Apple “iPad Mini,” which was released in 2012. Consider

(6) $$\begin{array}{c} \text{Rel}\left(t_{j},d_{j}\right)=\frac{\text{TagCount}\left(t_{j},d_{j}\right)}{\text{TotCount}\left(d_{j}\right)}. \end{array}$$

A comparison test was performed to compare the proposed method and the previous work [5]. The tag relevance measuring formula (6) is equivalent to Im's formula.

TagCount denotes the number of times tag(*t*_*j*_) is assigned to contents (*d*_*j*_). TotCount(*d*_*j*_) denotes the total number of tags assigned to the contents *d*_*j*_.

The content theme that was used for this experiment was Google Nexus 7, which is noted for Internet service, smartphone, and the notebook OS. A total of nine tags were posted, such as “Tablet,” “iPad,” “Android,” “Google,” “Nexus,” “Samsung,” “smartphone,” “Galaxy Tab,” and “Galaxy Note.” Among these, the “Tablet,” “Android,” “Google,” and “Nexus” tags were directly related to the theme of Figure 10. The rest had low correlations. The main contents of Figure 10 are related to the “Nexus 7” tablet PC. In addition, the “iPad” and “Galaxy Tab” tags were registered because they are classified as tablet PCs.  Table 2 shows that the frequency of the “Nexus” tag, which is related to the theme, and the “Samsung” tag, which has a low correlation with the theme, were given the same measurement values on two occasions. The measurement value of the tag relation in Im's study is the same as the Rel-method row in Figure 10.

When judging only by measuring the values from the existing method and suggested method, the degree of similarity of the two tags—“nexus” and “Samsung”—can be considered to be the same. However, the “Nexus” tag refers to a tablet PC that was released by Google and it should be considered as having high similarity with the Google-related tag. Methods that measure the relationship between the tag and the contents based simply on frequency have a problem due to the limitations in measuring the relevance between contents and tags.

On the other hand, the comparative result of the suggested method shows a weighted value of “0.0778” for the “Nexus” tag and a weighted value of “0.0579” for the “Samsung” tag. This proves that simple frequency measuring methods such as Im are not effective for distinguishing tags having little or no relation to the details of the contents. However, the method proposed in this study, which measures the synonym relevance and the synonym similarity distance, gives better judgments about the relevance of tags and contents.

### 5.2. Weighted Value Comparison between Correct Tagging and Entirely Incorrect Tagging

In paragraphs 2 and 3, for the performance testing of the suggested method, the experiment was conducted partially for valid tagging and invalid tagging of the contents.

**Figure 11:**
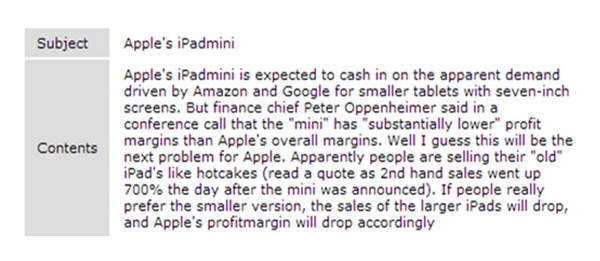
Contents for the “Apple iPad Mini” theme.

Figure 11 shows the contents that were written for the “Apple iPad Mini” theme. Apple is also noted for the Apple tablet product. Five tags, which were related to theme, were entered as shown in Table 3.

To compare the weighted values for valid tags and invalid tags, Table 4 shows tags of well-known overseas wear such as “Nike,” Gucci,” and “Levis.” The synonym analysis and similarity distance for each tag show different relation row based on brand names.

**Figure 12:**
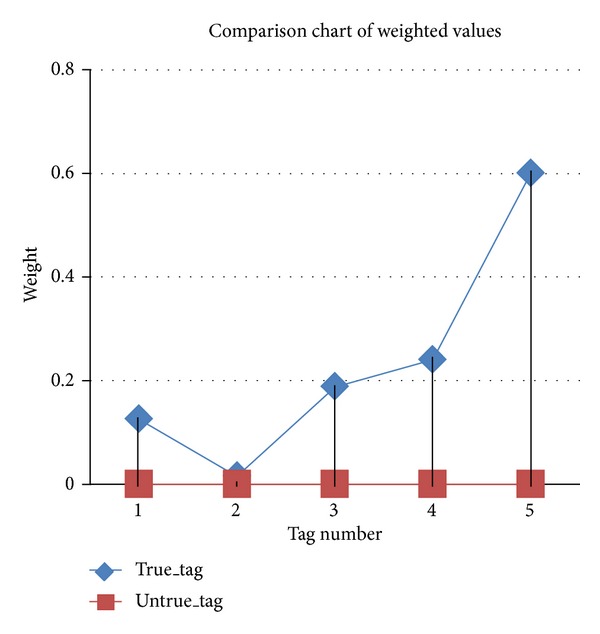
Comparison chart between “correct tagging” and “100% incorrect tagging.”

Figure 12 is a comparison chart of weighted values between valid and invalid tags. True_tag refers to an attempt to enter a valid tag and Untrue_tag refers to an attempt to do the opposite. It shows a maximum weighted value of “0.6” in the case of valid tagging and the highest weighted value for the Untrue_tag values is “levis”, which was “0.0032”. When the two tagging methods are compared, the weighted value shows a great difference.

### 5.3. Experimental Results and Analysis

This section deals with the question of how to differentiate the method used in basic research by complementing the following features.  Table 5 shows a qualitative comparison between the proposed method and other methods.

FolkRank, which was presented by Hotho et al. [8] with complementary additions to the existing PageRank method, is the leading algorithm among social tagging service algorithms that analyze the link relations for folksonomy using the graph-based approach. However, because it does not take the weight of the words included in a sentence or the weight of synonyms and their relevance into consideration, this ranking method fails to present ranking results in a detailed and accurate manner, by analyzing the relationship between users, tags, and resources. Another problem is that the user may intentionally write tags and expose advertisements for wrongful purposes. Therefore, it is necessary to extract precise keywords that represent the actual document and measure the similarity between document keywords and the tags in an exact way.

As a result, the measure of similarity based on the ranking measurement algorithm serves as an upgrade to the existing vector model and the FolkRank method. The proposed method showed improved outcomes as compared to the existing FolkRank method and the vector model. However, it is not possible to verify its efficiency and accuracy completely as criteria for standardized assessments have not been suggested.

## 6. Conclusion

With the popularization of Internet services and the rapid development in robotic applications, social web users have been overwhelmed and participated in many social media services that use collaborative tagging [15–18]. Collaborative tagging allows users to annotate the user-generated content and enables effective retrieval of uncategorized data [7]. Social tagging services allow Internet users to share web resources and they function as a foundation for ranking the collection of all resources and tags that have been created by users. Now that such social tagging services allow an enormous amount of information to be accumulated by many users in a short period of time and a growing number of users are intentionally abusing these services in an illegal manner in order to distort the ranking of specific resources.

As a result, users often have difficulties finding social media services that are matched to their needs [14]. While there exist some useful tags, useless and unwanted tags are also present in great numbers. This study found a solution that enables efficient and accurate ranking. In addition, refined social tags enable an analysis and classifications of effective social tagging services as they can go beyond merely classifying and managing contents to play a role as an important medium in information filtering. In the future, if the proposed method is utilized in the robotic applications, the efficient and accurate social tagging services using robotic recognitions can be realized.

## Figures and Tables

**Table 1:** Implementation environment.

Classification	Environment
Operation system	CentOS 6.3
Web server	Apache Web Server (2.2.9)
Database	MySQL
Language	PHP5, HTML, Javascript, and Ajax

**Table 2:** Comparison with an existing study and the proposed method.

Tag Number	Tag	Frequency	[10]	Proposed weight (*K*_*j*_)
1	Tablet	10	1.1111	0.5152
2	iPad	4	0.4444	0.1444
3	Android	3	0.3333	0.1222
4	**Nexus**	**2**	**0.2222**	**0.0778**
5	Google	2	0.2222	0.0650
6	**Samsung**	**2**	**0.2222**	**0.0579**
7	Galaxy Tab	1	0.1111	0.0261
8	Smartphone	0	0.0000	0.0042
9	Galaxy Note	0	0.0000	0.0050

**Table 3:** Results for valid tags on the “Apple iPad Mini” theme.

Tag Number	Tag	Frequency (*R*_*ij*_)	Total_tag (*T*_*i*_)	Relation (*S*_*j*_)	Distance (*D*_*j*_)	Weight (*K*_*j*_)
1	amazon	0.1	5	2.5	2.5	0.125
2	google	0.1	5	3	2.5	0.015
3	seven-inch	0.1	5	3.75	2.5	0.1875
4	ipadmini	0.1	5	8	1.5	0.24
5	apple	0.4	5	3.5	2.25	0.6

**Table 4:** Results for invalid tags on the “Apple iPad Mini” theme.

Tag Number	Tag	Frequency (*R*_*ij*_)	Total_tag (*T*_*i*_)	Relation (*S*_*j*_)	Distance (*D*_*j*_)	Weight (*K*_*j*_)
1	nike	0.001	5	4	2.5	0.0028
2	gucci	0.001	5	3.75	3	0.0026
3	levis	0.001	5	4	2.3333	0.0032
4	Versace	0.001	5	3.75	3	0.00262
5	Umbro	0.001	5	4	3.5	0.0028

**Table 5:** Comparison between FolkRank and the suggested method.

Variables	FolkRank	Our method
Ontologies	Not used	Used
Graph-based approach algorithm	Used	Used
Weight of the words	Not used	Used
Weight of synonyms and its relevance	Not used	Used
Prevention of manipulation	Medium	High
Efficiency of ranking	Medium	High
